# Anti-Inflammatory and Antioxidant Properties of *Physalis alkekengi* L. Extracts *In Vitro* and *In Vivo*: Potential Application for Skin Care

**DOI:** 10.1155/2022/7579572

**Published:** 2022-10-19

**Authors:** Bing-Wei He, Fei-Fei Wang, Li-Ping Qu

**Affiliations:** ^1^Yunnan Botanee Bio-technology Group Co., Ltd., Yunnan 650106, China; ^2^Yunnan Yunke Characteristic Plant Extraction Laboratory Co., Ltd., Yunnan 650106, China; ^3^Shanghai Jiyan Bio-pharmaceutical Co., Ltd., Shanghai 201702, China

## Abstract

**Objective:**

Inflammatory skin disorders are becoming major issues threatening public health with increasing prevalence. This study was to evaluate the anti-inflammatory, antioxidant, and antisenescent activities of traditional folk medicinal plant, *Physalis alkekengi* L. extracts to alleviate skin inflammation and its possible mechanisms.

**Methods:**

Lipopolysaccharides (LPS)-treated murine macrophages RAW264.7 and human skin keratinocytes HaCaT were incubated with the plant extracts, respectively. The production of nitric oxide (NO) was tested by using Griess reagents. The activity of nitric oxide synthase (NOS) was detected through a fluorescence microplate reader. Reactive oxygen species (ROS) production and cell apoptosis were quantified by flow cytometry. The proinflammatory cytokines were measured using ELISA and qRT-PCR. Human skin fibroblasts (HFF-1) were coincubated with D-galactose (D-gal) and the plant extracts. The senescence associated-galactosidase (SA-*β*-gal) was stained to evaluate cellular senescence. The senescence-associated secretory phenotype (SASP), IL-1*β,* was measured through ELISA. The mRNA of IL-1*α* in SLS-stimulated and PGE2 in UV-radiated 3D skin models were detected by qRT-PCR. *In vivo* ROS production and neutrophil recruitment in CuSO_4_-treated zebrafish models were observed by fluorescence microscopy. Inflammation-related factors were measured by qRT-PCR*. Results*. *In vitro*, *Physalis alkekengi* L. significantly reduced NO production, NOS activity, cell apoptosis, transcription of TNF-*α*, IL-6, IL-1*β* and ROS production. These plant extracts markedly attenuated SA-*β*-gal and IL-1*β* and downregulated the production of IL-1*α* and PGE2. *In vivo*, the plant extracts dramatically dampened ROS production, the number of neutrophils, and proinflammatory cytokines.

**Conclusions:**

Cumulatively, this work systematically demonstrated the anti-inflammatory, antioxidant, and antisenescent properties of *Physalis alkekengi* L. and proposed the possible roles of *Physalis alkekengi* L. in inflammatory signaling pathways, providing an effective natural product for the treatment of inflammatory skin disorders.

## 1. Introduction

The human derma is the biggest, most extensive organ of the body that covers around 1–2 m^2^ area [1,2]. Derma not only regulates body temperature, and feels the touching, but also defends against the invasion of external stimuli [3]. Microscopically, the human derma appears to be orderly composed of epidermis, epidermis-dermis junction, dermis, and hypodermis from the outside in. Keratinocytes predominate in the epidermis in a well-defined manner and produce keratin associated with desmosomes to protect the skin from external attacks. The dermis includes abundant fibroblasts that release collagen and elastic fibers to the extracellular matrix (ECM) for the plasticity of skin [4, 5]. Nowadays, skin health attracts more and more people's attention with the improvement of life quality [6]. Genetic (predisposition) [7] and environmental factors (UV-radiation [8], chemical and contagious agents exposure [9, 10], and heat/cold exposure [11]) play important roles in most allergic skin diseases such as atopic dermatitis [12], contact dermatitis [13, 14] and urticarial. Most of these allergic skin disorders are chronic, inflammatory, and proliferative [15]. The skin usually turns red, pigmented, pruritic, dry, and cracked [16] during disease development. Moreover, the interaction between chronic inflammation and senescence is attracting increasing attention [17–20]. In addition to allergic skin diseases, inflammation is closely related to pathophysiological processes in various inflammation-associated diseases such as atherosclerosis [21], acute lung injury (ALI) [22], chronic obstructive pulmonary disease (COPD) [23], periodontal disease [24] and inflammatory bowel disease (IBD) [25], which bound tightly to one another. These symptoms might deteriorate the preexisting inflammation diseases. Thus, suppressing the occurrence of skin inflammation seems to be an efficient way to relieve skin allergy, skin senescence, and inflammation-related illnesses. Hence, looking for high-performance anti-inflammatory ingredients for drugs or skincare products is considered to be a promising strategy for preventing skin diseases, aging, and inflammation-associated disorders.

Traditional natural plants are not only used as food to satisfy human necessities but also as medical and cosmeceutical ingredients [26, 27]. A huge amount of studies report numerous constituents of plant extracts possess outstanding activities such as anti-inflammation [28, 29], and antioxidation [30, 31] *in vitro* and *in vivo*. For instance, extracts of *Physalis alkekengi* L. [32–35], *Artemisia princeps* Pamp. [36], and *Cannabis sativa* L. [37] exhibit anti-inflammatory properties *in vitro* while extracts of Antarctic Lichen (*Umbilicaria antarctica*) [38], Soft Coral (*Dendronephthya puetteri*) [39], and *Forsythia suspensa* (Thunb.) Vahl [40] exert their anti-inflammatory abilities *in vivo* as well. Currently, the safety of plants gradually attracts the wide attention of scientists and consumers which could explain the popularity of natural plant extracts as functional ingredients for medicinal drugs and skincare products. Based on a large quantity of research, the total phenolic and flavonoid contents among plant extracts were identified as the functional constituents [28, 41–43]. For example, flavonoids luteolin and luteolin-7-O-glucoside derived from dandelion flowers strengthen the anti-inflammatory property and antioxidant properties *in vitro* [44].

Genus *Physalis* belongs to the Solanaceae family, distributed in Mexico, Europe, and South and Central Asia, including about 120 species [45]. Among them, *Physalis alkekengi* L. widely distributed in China [46, 47] is popular both in food and folk medicine as an expectorant, antitussive, diuretic, and oxytocic agents. The fruit calyces and roots of *Physalis alkekengi* L. used as an analgesic, antibacterial drugs, etc. in folk medicine have been reported [35]. Besides, many other pharmacological properties, including antimicrobial [48], antioxidation [49], antiapoptosis, antihyperglycemia [50], and amelioration of the side effect of cisplatin-induced nephrotoxicity [51] *in vitro* and *in vivo* have been studied. 16,24-cyclo-13,14-secosteroids named physalins have been isolated from the fruit calyces of *Physalis alkekengi* L. and identified since 1970 [52]. After that, dozens of physalins have been isolated [53–56]. In addition, minor metabolites such as alkaloids and flavonoids have also been identified in different parts of the plant [47, 57]. The fruit calyces of *Physalis alkekengi* L. extracts (PEs) mainly contain the functional components physalins and flavonoids such as luteolin-7,4′-di-O-*β*-D-glucopyranoside which exhibit excellent anti-inflammatory activity in lipopolysaccharide (LPS)-induced macrophages RAW264.7 [32]. However, studies on the anti-inflammatory and antisenescent properties of PEs in human skin cells are scarce.

In this work, we collected PEs and standardized the content of flavonoids (luteolin-7,4′-di-O-*β*-D-glucopyranoside). The anti-inflammatory properties of PEs were tested *in vitro* and *in vivo* in two different inflammation patterns (LPS-induced and CuSO_4_-stimulated). Levels of inflammation-associated cytokines and enzymes were evaluated not only in macrophages but also in skin cells to broaden the cosmeceutical application of PEs. Moreover, the antiaging effect of PEs was tentatively explored at a cellular level through the D-galactose (D-gal) and H_2_O_2_-induced fibroblast aging model to confirm the crosstalk between inflammation and cellular senescence. At last, both the anti-inflammatory and antioxidant capabilities of PEs were estimated in zebrafish embryos. The robust results of PEs *in vitro* and *in vivo* demonstrated the potential benefits of PEs for skin care.

## 2. Materials and Methods

### 2.1. Chemical

D-galactose (D-gal), lipopolysaccharide (LPS), sodium lauryl sulfate (SLS), 2′,7′-dichlorodihydrofluorescein diacetate (H2DCFDA), copper sulfate pentahydrate (CuSO4·5H2O) were purchased from Sigma-Aldrich (St. Louis, MO, USA). Cell-countingkit-8 (CCK-8) was obtained from Dojindo Laboratories (Japan). Quantitative real-time polymerase chain reaction (qRT-PCR) primers were purchased from Generay Biotech Co., Ltd (Shanghai, CHINA). GeneJET RNA purification kit was bought from Thermo Fisher Scientific (USA). RNA Reverse Transcription Kit was obtained from PrimeScript™ RT reagent Kit (Takara, Japan). ELISA kits were bought from NeoBioscience (Xinbosheng, China). NO assay kit, NOS kit, LDH assay kit, *β*-gal staining kit, and Annexin V-FITC/PI apoptosis double staining kit were purchased from Beyotime (Jiangsu, China). All chemicals were used as received without further purification.

### 2.2. Plant Material and Extracts Preparations

**Figure 1:**
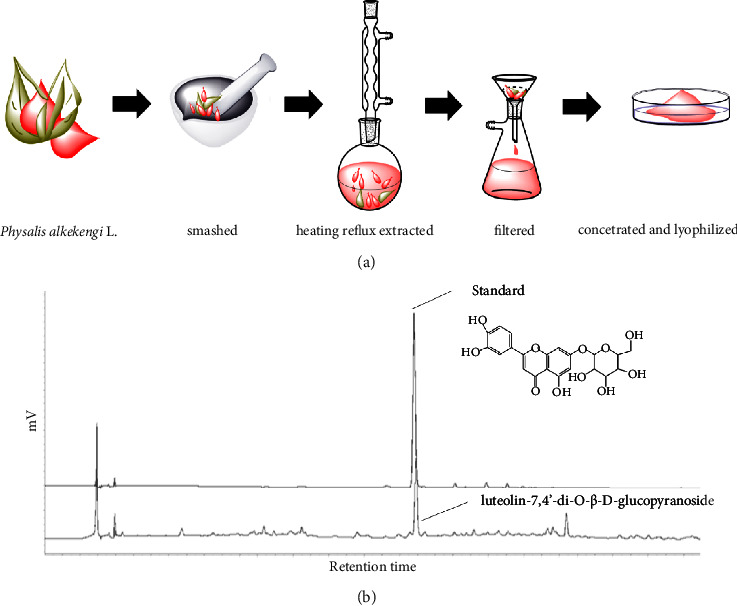
Flow chart for the extraction of *Physalis alkekengi* L. (a) and the luteolin-7,4′-di-O-*β*-D-glucopyranoside analyzed by HPLC (b).

*Physalis alkekengi* L. was collected from Kunming, Yunnan Province, the People's Republic of China in November 2018 and identified by Professor Haiyang Liu (based on permission No. 2018–11 from Kunming Institute of Botany, Kunming, China). *Physalis alkekengi* L. has been deposited in voucher specimen (No. 20181111) in the herbarium of Botany Research Institute, Shanghai Jiyan Bio-Pharmaceutical Development Co. Ltd., Shanghai, China. The part of the plant to be analyzed was thoroughly rinsed in distilled water and dried at room temperature. Collection of the fruit calyces from *Physalis alkekengi* L., the organization of experimental research and field research for plants (cultural or wild) was carried out in accordance with the Environmental Code of the People's Republic of China dated April 24, 2014. The dried persistent fruit calyces of *Physalis alkekengi* L. were smashed into small pieces and put into a 250 mL flask with subsequent 95% ethanol heating reflux extraction for 2 h at 80°C. The residue was extracted another two times by the same procedure. The total extracts were collected and filtered to get rid of the undissolved residue. The combined solution was concentrated in a vacuum rotary evaporator and lyophilized immediately to obtain PEs powder. The crude extracts were stored at −80°C for the following experiments. The process of extraction was simply shown in Figure 1(a). For tested samples, PEs were dissolved in DMSO (dimethyl sulfoxide) to make a stock solution of 200 mg/mL. The stock solution was diluted using the cell culture medium for subsequent experiments *in vitro* and *in vivo*.

### 2.3. Analysis of Bioactive Compounds of PEs

The main bioactive compounds of PEs include bitter flavors and flavonoids. The extracts were diluted to 5 mg/mL using distilled water and analyzed by high-performance liquid chromatography (HPLC) with a referred claim in Chinese Pharmacopoeia. The effective content of luteolin-7,4′-di-O-*β*-D-glucopyranoside in PEs should be more than 0.42%, at which PEs exerts inflammatory activity.

### 2.4. Cell Culture

Immortal human keratinocyte (HaCaT), human skin fibroblast (HFF-1), and murine macrophages (RAW264.7) were bought from the Cell Bank of the Chinese Academy of Science (Shanghai, China). Cells were cultured in Dulbecco's Modified Eagle's Medium (DMEM) supplemented with 10% (v/v) fetal bovine serum at 37°C, 0.5% CO_2_. HaCaT and HFF-1 were digested with 0.25% trypsin while the RAW264.7 cell line was passaged without any trypsin treatment.

### 2.5. Cell Viability

(a) 2 × 10^4^ cells per well were seeded in a 96-well culture plate to reach approximately 80% confluence. No-cell wells were regarded as positive controls (PC). The culture medium was replaced by another 100 *μ*L of complete culture medium containing different content of PEs for 24 h, and nontreatment wells were as negative controls (NC). PEs solution was substituted with 100 *μ*L of 10% CCK-8 solution for another 2 h incubation followed by an absorbance reading taken at 450 nm. The cell survival was calculated according to the following formula: cell viability (%) = (OD_sample_ - OD_pc_)/(OD_nc_ - OD_pc_) × 100%. Five repeats were conducted for each condition. (b) The lactate dehydrogenase (LDH) activity of cells was evaluated via an LDH assay kit. 1 × 10^5^ cells/well were cultured in a 24-well plate for 24 h to achieve around 80% confluence, then either treated with PEs solution or not. PC was treated with the same volume of PEs-free DMEM. After 2 h incubation, H_2_O_2_ at 800 *µ*M was substituted for both PEs solution and PEs-free culture medium for 2 h incubation. Then, 500 *µ*L of complete medium replaced H_2_O_2_ and incubated for another 20 h. After, 200 *µ*L of supernatant alone was harvested and mixed with 20 *µ*L of releasing agent while the 200 *µ*L of PC medium together with cells were incubated with the equivalent releasing agent. 200 *µ*L of DMEM alone mixed with 20 *µ*L of releasing agent was as NC. Then, the solution was incubated for 1 h at 37°C, 5% CO2 and centrifuged at 1000 rpm for 4 min. 40 *µ*L suspension per well was collected into a 96-well plate and mixed with 20 *µ*L fresh LDH working solution that was prepared according to the manufacturer's protocol. The plate was vortexed in the dark for 30 min and the OD values were measured at 490 nm wavelength. LDH release (%) was expressed via the following equation: LDH (%) = (OD_sample_ - OD_nc_)/(OD_pc_ - OD_nc_) × 100%. Triplicate was performed for each group.

### 2.6. Cell Apoptosis Assay

The percentage of early-stage apoptosis and lately-stage apoptosis, necrosis of cells induced by the addition of LPS or LPS/PEs mixture were determined through Annexin V-FITC/PI double staining assay kit (Beyotime). Cells were seeded and treated with PEs solutions together with 1 *µ*g/mL of LPS or LPS alone. Nontreatment wells were as controls. After incubation of PEs mixed with LPS for 24 h, suspended medium (200 *µ*L) and trypsin-disassociated cells were collected and centrifuged. The precipitate was resuspended in 195 *µ*L of binding buffer plus 5 *µ*L of Annexin V-FITC for 5-min incubation followed by 10-min incubation of 10 *µ*L Propidium Iodide (PI). The nonstaining sample was as a reference. After a gentle mix at room temperature and a dark environment was conducted, the percentage of cell death was detected via Flow Cytometry (FCM). The cell plot was distributed into four quadrants based on Annexin V-FITC and PI axes representing living cells in quadrant 3, early-stage apoptotic cells in quadrant 4, and total lately-stage apoptotic and necrotic cells in quadrant 1, respectively.

### 2.7. Clearance of Nitric Oxide (NO) Production

2 × 10^4^ cells/well were seeded in a 96-well plate and incubated for 24 h. The culture medium was substituted by 100 *µ*L of PEs solution at different concentrations or 10 *µ*g/mL of Dexamethasone (Dex) as a positive control for 1 h, and then LPS was added to the medium directly to achieve 1 *µ*g/mL for another 24 h incubation. The content of NO in the supernatants was detected according to the manufacturer's protocol of the Total Nitric Oxide assay kit (Beyotime).

### 2.8. Nitric Oxide Synthase (NOS) Assay

The activity of total NOS was detected through the NOS assay kit (Beyotime). Nonfluorescent and cell-permeable DAF-DA was esterified to weakly fluorescent DAF-FM which reacted with NO to yield fluorescent benzotriazole derivative. 2 × 10^4^ RAW264.7 cells per well were cultured in a 96-well plate for 24 h and treated with PEs mixed with LPS for another 24 h as similar to the assay of NO production. After, the medium was replaced by 100 *µ*L of test buffer with a subsequent 100 *µ*L of the reaction solution. The solution mixture was cultured continuously for another 30 min. The fluorescence intensity was detected at 495 nm excitation and 515 nm emission. The Relative Fluorescence Unit (RFU) of NOS which reflects the activity of NOS was determined as the following equation: RFU of NOS ＝ (RFU_treatment_ - RFU_blank_)/(RFU_nontreatment_ - RFU_blank_) where “RFU_treatment_” means the fluorescence intensity of cells treated by PEs and LPS or LPS alone, “RFU_nontreatment_” means the fluorescence intensity of 1 × 10^5^ cells/well without treatment, “RFU_blank_” represents the fluorescence intensity of buffers alone.

### 2.9. Enzyme-Linked Immunosorbent Assay (ELISA)

1 × 10^5^ cells per well were seeded in 24-well for 24 h. For HFF-1, cells were pretreated with 500 *µ*L of PEs solution at different concentrations for 2 h, then PEs solution was replaced by 400 *µ*M of H_2_O_2_ for 2 h. After, cells were incubated with a freshly complete medium for another 20 h. For RAW264.7, LPS-induction was conducted according to the method of nitric oxide (NO) assay for 24 h. For HaCaT, cells were incubated with PEs at different concentrations for 12 h, then LPS was added to achieve the concentration of 10 *µ*g/mL for another 12 h incubation. Inflammatory-associated cytokines in the supernatant were detected according to the procedure of the ELISA kit (Xinbosheng, China).

### 2.10. Quantitative Real-Time Polymerase Chain Reaction (qRT-PCR)

2 × 10^5^ cells/well were seeded in a 12-well plate for 24 h. Cells were treated with LPS according to the method of ELISA. The supernatant was collected for ELISA assay while the mRNA of cell precipitate was extracted via GeneJET RNA purification kit (Thermo Scientific). The total mRNA was quantified by NanoDrop one (Thermo Scientific) and reversely transcripted using PrimeScript RT Master Mix (Takara, Shiga, Japan) according to the manufacturer's protocol (500 *µ*g RNA was reversely transcripted to cDNA in a final volume of 10 *µ*L). qRT-PCR was performed in a total volume of 20 *µ*L including 10 *µ*L of SYBR Green PCR Master Mix (Roche), 2 *µ*L of cDNA template, and 0.4 *µ*L of each primer (10 *µ*M) with a LightCycler 96 instrument (Roche). The standard cycling conditions were 95°C for 10 min followed by 40 cycles of 95°C for 30 s and 60°C for 30 s. The ratios of the number of candidate genes to the housekeeping gene were analyzed using LightCycle 96 software to evaluate the relative mRNA levels of candidates. The sequences of primers were listed in Supplementary Table S1. Each condition was performed in triplicates.

### 2.11. Antioxidant Activity of PEs

1 × 10^5^ HaCaT cells per well were seeded in a 24-well plate for 24 h and pretreated with PEs solutions for 2 h prior to 800 *µ*M of H_2_O_2_ for another 2 h. After, the level of reactive oxygen species (ROS) was quantified immediately using a ROS detection kit (Beyotime) and FCM. Briefly, cells were washed with serum-free DMEM and incubated with 1000 × diluted DCFH-DA which was hydrolyzed to nonfluorescent DCFH inside the cell. ROS oxidized nonfluorescent DCFH to fluorescent DCF. The relative ROS level could be represented by the relative concentration of DCF through the fluorescence intensity of DCF detected by FCM.

### 2.12. Antisenescent Activity of PEs

Senescence-associated-*β*-galactosidase (SA-*β*-gal) activity is one of the biomarkers of cellular senescence. 5 × 10^4^ HFF-1 cells were seeded in a 24-well plate for 24 h and incubated with PEs solution at different concentrations together with 20 mg/mL D-gal for 72 h. The enzymatic activity of cytosolic SA-*β*-gal was determined by in site X-gal staining kit (Beyotime). Simply, cells were washed twice with PBS and fixed for 10 min at room temperature. Then cells were washed with PBS three times (each 3 min), followed by overnight incubation of the working solution containing the substrate X-gal at 37°C in the absence of CO_2_. A parafilm was used to prevent working solutions from evaporation. After, cells were further washed by PBS once and observed through an optical microscope. The number of *β*-gal positive cells represented the degree of cell senescence.

### 2.13. Anti-Inflammatory Effect of PEs on a 3D Skin Model

The commercial epidermal tissue EpiSkin (L'Oréal Research and Innovation Center, Shanghai, China) is an *in vitro* reconstructed human epidermis (RHE) from normal human keratinocytes cultured on a collagen matrix at the air-liquid interface. Thus, RHE is histologically similar to the *in vivo* human *epidermis* [58]. The EpiSkin models were removed from the nutrient agar and equilibrated in 12-well plates at 37°C, 5.0% CO_2_ overnight. EpiSkin was irritated by 0.3% SLS, then incubated with 1 mg/mL PEs for another 20 h, nonirritated skin but still treated with PEs was a negative control. The concentration of proinflammatory factor IL-1*α* in the supernatant was detected by ELISA as described above. To get the UV-induced skin model, EpiSkin was exposed to the 30 J/m^3^ UV for 2 h, followed by treatment of PEs for another 20 h. EpiSkin treated with PEs but not exposed to UV was used as a negative control. The content of prostaglandin E2 (PGE2) in the culture medium was measured by ELISA.

### 2.14. Toxicity of PEs in Zebrafish

The following animal experiments were reviewed and approved by The animal care and use committee of Changzhou University (ACUC NO: 20180201). We declared that all experiments and methods for the zebrafish model were performed in accordance with the internationally accepted principles for laboratory animal use and care “Animal Research: Reporting of In Vivo Experiments (ARRIVE)” guidelines. Zebrafish experiments were conducted in line with the relevant laws and the guidelines issued by the Ethical Committee. Three days postfertilization (3-dpf) zebrafish embryos were picked up at a density of ten embryos per well and incubated with PEs at different doses (0.1, 1, 10, 30, 100, 300 *µ*g/mL) for 24 h at 28°C. The percent survival of zebrafish was directly determined through observation under bright-field microscopy.

### 2.15. Antioxidant Effect of PEs on Zebrafish

Quantification of ROS in Zebrafish was determined by the cell-permeable fluorescent probe H2DCFDA. H2DCFDA is hydrolyzed inside the cell to nonfluorescent probe CM-H2DCFDA which could be oxidized by ROS to fluorescent dye DCF. The fluorescence intensity of DCF reflects the oxidative stress of the cell. Thirty zebrafish embryos (3-dpf) per group were selected and treated either with PEs at different concentrations (30, 100 *µ*g/mL) or not for 24 h at 28°C. Zebrafish embryos were washed twice and incubated with 20 *µ*g/mL H2DCFDA for 1 h in the dark. After, embryos were anesthetized with 0.0003% MS-222 (tricaine methane-sulfonate) and subsequently observed through a fluorescence microscope. The average fluorescent intensity of the individual group was quantified using SpectraMax i3x Multimode microplate reader and calculated through Image J. Group treated with 3 mg/mL of Tripeptide-1 was used as a positive control (PC).

### 2.16. Anti-Inflammatory Effect on Zebrafish

Ten 3-dpf transgenic zebrafish embryos with neutrophil-specific fluorescence: Tg (mpx: dsRED) were chosen and exposed to 1 *μ*M of CuSO_4_ either alone or together with 30 or 100 *µ*g/mL PEs for 2 h at 28°C. The embryos without CuSO_4_ treatment were used as a negative control. After the development and fluorescence intensity of zebrafish embryos were observed by fluorescence microscope, in the meanwhile, the number of neutrophils was counted by image J. Anti-inflammatory activity of PEs was represented by the reduced percentage of neutrophils.

### 2.17. qRT-PCR in Zebrafish

Thirty 3-dpf zebrafish embryos per group were stimulated by 1 *μ*M CuSO_4_ as written in the method “Anti-inflammatory effect on zebrafish”. The total RNA was extracted from zebrafish embryos through RNA-Quick Purification Kit (ES Science) following the manufacturer's instructions. RNA was dissolved and reverse-transcribed into cDNA. The following qRT-PCR for the measurement of five oxidation- and inflammation-associated genes expression were conducted as described in the above method of qRT-PCR. The details of primers were given in Supplementary Table S2. All conditions were performed in triplicates.

### 2.18. Data Statistics

Data were represented as the mean ± standard of at least two independent repeated experiments done in duplicate or triplicate. Differences were considered at NS, *p* > 0.05, ^*∗*^*p* < 0.05, ^*∗*^^*∗*^*p* < 0.01, ^*∗*^^*∗∗*^*p* < 0.001, ^#^*p* < 0.05, ^##^*p* < 0.01, and ^###^*p* < 0.001 using a two-tailed Student's t-test.

## 3. Results and Discussion

### 3.1. Quantification of Luteolin-7,4′-di-O-*β*-D-glucopyranoside in PEs

The fruit calyces of *Physalis alkekengi* L. were extracted according to the procedure described in Figure 1(a). Lyophilized PEs were dissolved in DMSO and analyzed by high-performance liquid chromatography (HPLC). The highest peak in Figure 1(b) was verified according to the retention time and a spike with a standard of luteolin-7,4‘-di-O-*β*-D-glucopyranoside. The effective peak area was completely separated from other compositions and reached 0.42%, which was well suited to the claim of Chinese Pharmacopoeia.

### 3.2. Anti-Inflammatory Property of PEs in LPS-Stimulated RAW264.7 Cells

#### 3.2.1. Inhibition of NO and Nitric Oxide Synthase (NOS) Production by PEs in LPS-Stimulated RAW264.7 Cells

**Figure 2:**
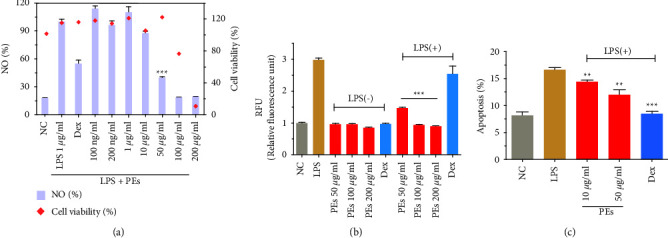
Quantification of NO production and cell viability (a), NOS activities (b), and cell apoptosis (c) in RAW264.7. PEs at different concentrations were incubated with cells for 1 h prior to incubation with LPS (1 *μ*g/mL) for 24 h. Treatment of LPS plus Dex at 10 *μ*g/mL was a positive control. Each experiment was conducted in triplicate. Significant differences from the LPS-treated group were regarded as  ^*∗∗*^*p* < 0.01,  ^*∗∗∗*^*p* < 0.001.

The effective concentrations of PEs were determined through a well-known model of LPS-induced inflammation in RAW264.7 murine macrophage. LPS is a major component of the Gram-negative bacterial cell wall that induces inflammation through known TLR4-MyD88 signaling [59, 60]. The inflammatory repertoire of the culture medium is initially evaluated with regard to the level of NO production.  Figure 2(a) showed that 10 *µ*g/mL of PEs slightly eliminates the formation of NO while 50 *µ*g/mL of PEs significantly attenuates 60% of NO production, exceeding that of positive control dexamethasone (Dex). Besides, noncytotoxicity of PEs below 100 *µ*g/mL was observed. We subsequently studied the total NOS activity of PEs at the effective concentrations of 50, 100, and 200 *µ*g/mL.  Figure 2(b) suggested PEs entirely suppressed the enzyme activities while Dex only had a slight inhibition of NOS activity. These results elucidated that PEs exhibited excellent anti-inflammatory properties *in vitro*. LPS-stimulated inflammation could further contribute to cell death. Since PEs shows great potential for anti-inflammatory agents, we would like to explore the protection of PEs from cell apoptosis. As Figure 2(c) showed, PEs presented a good protective effect against LPS-induced apoptosis in a dose-dependent way.

#### 3.2.2. Inhibition of Differentiation in LPS-Stimulated RAW264.7 Cells

We then examined the protection of PEs against LPS-induced morphology changes by using microscopy. The bright-field microscope images in Figure S1 indicated that 1 *µ*g/mL LPS induced the small round-shaped RAW264.7 into an elongated shape of dendritic filopodia, while both 50 *µ*g/mL and 100 *µ*g/mL PEs limited cell differentiation. In addition, 100 *µ*g/mL PEs alone did not make any influence on the cell compared to the group of NC.

#### 3.2.3. Anti-Inflammatory Mechanism of PEs

**Figure 3:**
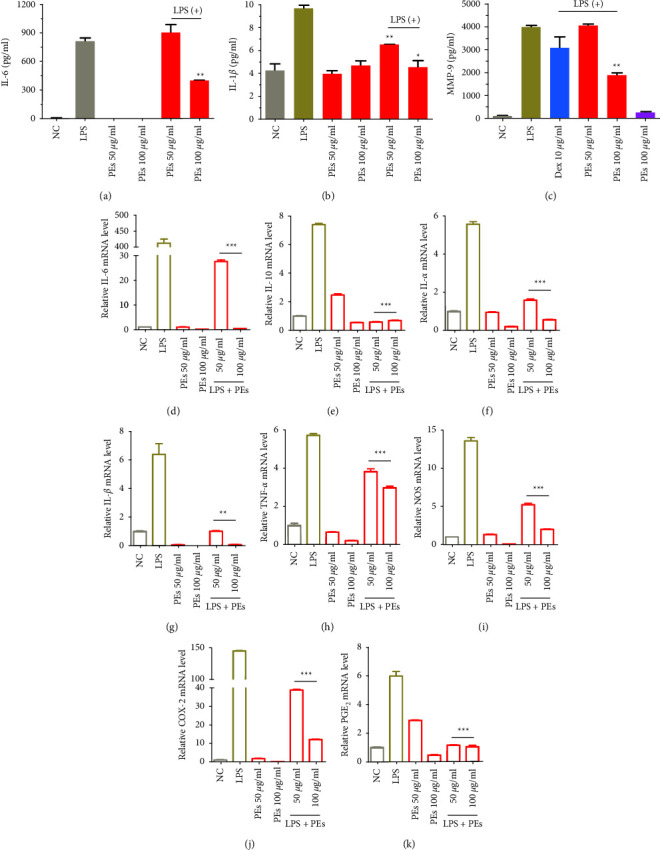
Anti-inflammatory effects of PEs on LPS-stimulated RAW264.7. Cells were pretreated with PEs at the concentrations of 50, 100 *μ*g/mL for 1 h and exposed to 1 *µ*g/mL LPS for another 24 h. After, IL-6 (a), IL-1*β* (b), and MMP-9 (c) production in the cell culture supernatant were detected by using an enzyme-linked immunosorbent assay (ELISA). The cells were collected and lysed for the measurement of the relative expression of genes involved in immune responses (IL-6 (d), IL-10 (e), IL-*α* (f), IL-1*β* (g), TNF-*α* (h), NOS (i), COX-2 (j), and PGE2 (k)) by qRT-PCR analysis (*β*-Actin expression was used as an internal control). Each experiment was performed in triplicate. ^*∗*^*p* < 0.05,  ^*∗∗*^*p* < 0.01,  ^*∗∗∗*^*p* < 0.001 compared to the group of LPS-treatment alone.

To further explore the anti-inflammatory mechanism involved in the treatment of PEs, we investigated the expression levels of two well-known proinflammatory factors interleukins (ILs) IL-6 and IL-1*β *cell-secreted in the supernatant of LPS-induced RAW264.7. The plot in Figure 3(a) indicated that LPS-induced 800-fold-higher levels of IL-6 production than that in the negative control, 100 *µ*g/mL of PEs inhibited approximately 50% of LPS-induced IL-6 expression. Similarly, Figure 3(b) suggested that 100 *µ*g/mL PEs totally down-regulated the expression of IL-1*β* to a normal level. By the way, treatment of PEs alone would not make any effect on the proinflammatory factors production. Inflammation and cellular senescence have been connected directly through ILs-dependent inflammatory networks [61]. Taking into consideration that ILs production was greatly inhibited by PEs above, PEs may take part in antisenescence as well. Matrix metalloproteinase-9 (MMP-9) is expressed in the intracellular and extracellular matrix, maintaining the construction of intercellular collagen and fibers. Moreover, MMP-9 belonging to the member of senescence-associated secretory phenotypes (SASPs) plays a role not only in the signaling pathway of inflammation but also in cell senescence.  Figure 3(c) showed that LPS extremely promoted the expression of MMP-9 while 100 *µ*g/mL PEs dramatically reduced 50% of MMP-9 expression. These results further revealed the anti-inflammatory properties and the antiaging potential of PEs. Furthermore, we investigated the mRNA levels of inflammation-related factors through high-sensitivereal-time fluorescent quantitative PCR (qRT-PCR). In Figure 3(d)–3(k), LPS-stimulation resulted in a significant increase in the transcription activities of several proinflammatory cytokines (IL-6, IL-10, IL-*α*, and IL-1*β*) and TNF-*α* mRNA, while 50–100 *µ*g/mL of PEs performed a significant decrease in the levels of them. That provided more evidence for the anti-inflammatory effect of PEs. Moreover, gene levels of important mediators of inflammation, NOS, cyclooxygenase-2 (COX-2), and the metabolic prostaglandin E2 (PGE2) were down-regulated through PEs incubation compared with non-PEs incubation in response to LPS-stimulation. The details of PCR primer pair sequences were given in Supplementary Information Table S1.

### 3.3. Effects of PEs on Human Keratinocyte (HaCaT)

#### 3.3.1. Alleviation of LPS-Induced Cytotoxicity by PEs in Human Keratinocyte HaCaT

The anti-inflammatory property of PEs has been demonstrated in the LPS-induced inflammatory model of murine macrophages. Thus, we explored whether PEs could be used in skincare products since sensitive skin could secret large quantities of proinflammatory factors when encountering allergens. To investigate the protective ability of PEs against LPS-induced injury in human epidermal cells HaCaT, we studied the cytotoxicity of PEs at different concentrations for 12 h, 24 h, and 36 h incubation, respectively. As shown in Supporting Information Figure S2, PEs at concentrations of up to 100 ng/mL caused no cytotoxicity within 24 h incubation while showing non-negligible toxicity at 36 h incubation in HaCaT. Bright-field microscopy images of cell morphological changes treated by PEs in Figure S3(a) confirmed the safety of PEs incubation at concentrations below 100 ng/mL for 24 h. We further analyzed the protective effect of PEs at 25, 50, and 100 ng/mL.  Figure S3(b) indicated that PEs concentration-dependently reduced the LPS-induced cell damage.

#### 3.3.2. Anti-Inflammation of PEs in Human Keratinocyte HaCaT

Additionally, PEs exerted anti-inflammatory activity in HaCaT skin cells as well. Consequently, PEs at the range of concentrations (25–100 ng/mL) down-regulated the mRNA levels of inflammatory-related factors TNF-*α*, IL-6, and IL-1*β* shown in Figure S4 which further indicated the anti-inflammatory function of PEs and implied the possibilities of PEs for allergic skincare products.

#### 3.3.3. Antioxidant Activity of PEs in Human Keratinocyte HaCaT

The relationship between inflammation and oxidative stress is interdependent [62]. Oxidative stress develops in the site of inflammation due to the production of reactive species (ROS) and further promotes inflammation, while the secretion of proinflammatory factors eventually accentuates oxidative stress. Inhibition of ROS could be a way to attenuate inflammation. We evaluated the antioxidant properties of PEs in HaCaT cells through quantitative measurement of ROS formation. Here, the oxidative damage model was implemented through a well-established H_2_O_2_-induced cell injury [63]. The result in Figure S5(a) showed PEs at concentrations higher than 500 ng/mL could effectively reduce H_2_O_2_-induced oxidative stress. The protective effect of PEs from H_2_O_2_-induced damage has also been proven by endogenous lactate dehydrogenase (LDH) release assay in Figure S5(b), 100 ng/mL of PEs could prevent around 15% of H_2_O_2_-induced LDH release. Taken together, PEs exhibited both anti-inflammatory and antioxidant properties in HaCaT.

### 3.4. Anti-senescent Activity of PEs in Human Skin Fibroblast (HFF-1)

**Figure 4:**
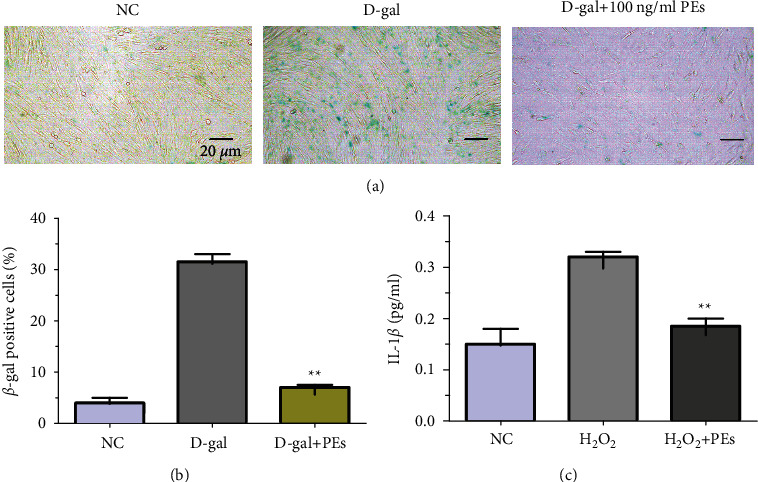
Antisenescent effect of PEs on HFF-1. Cells were treated with 20 mg/mL of D-gal together with 100 ng/mL of PEs for 72 h. After, cells were stained according to the manufacturer's protocol. *β*-gal positive cells (in blue) were observed through microscopy (scale bar represents 20 *µ*m) (a) and counted (b), moreover, the anti-inflammatory effect of PEs on HFF-1 was evaluated according to the ELISA experiment of IL-1*β* in cell culture medium after treatment with 400 *µ*M of H_2_O_2_ with/without 100 ng/mL PEs (c). Each experiment was performed in triplicate. ^*∗*^^*∗*^*p* < 0.01 compared to the groups of D-gal- or H_2_O_2_-treatment.

The excellent performance of PEs in anti-inflammation in RAW264.7 and oxidation-resistance in keratinocytes HaCaT shed new light on the role of PEs in antisenescence treatments since a number of antiaging ingredients exhibit well antioxidant activity. We explored the antiaging ability of PEs in the second passage of infant foreskin fibroblast HFF-1. Senescence-associated*β*-galactosidase (SA-*β*-gal) is the most widely used biomarker of aging which could be overexpressed in senescent cells and diseased tissues [64]. D-gal induces rat or cell-aged models which is similar to natural aging for the study of senescent mechanism [65, 66]. D-gal was first used in an aging inducer by Chinese researchers in 1985. It was reported that injections of D-gal shorten the longevity in mice [65], drosophila [67], and human fetal lung fibroblast [68]. Although the exact aging mechanism of D-gal remains unclear, the possible mechanism is that, on the one hand, D-gal is a reducing sugar that exists naturally in the body, however, excessive D-gal could be oxidized into aldose and hydrogen peroxide through galactose oxidase, leading to the production of ROS [69] which subsequently cause oxidative stress, senescence, inflammation and apoptosis [70]; on the other hand, D-gal reacts with the free amines of amino acids in proteins and peptides to form advanced glycation endproducts (AGEs) which are associated with senescence, diabetes, Alzheimer's disease [65].  Figure 4(a) showed treatment only with D-gal extremely increased the percentage of SA-*β*-gal positive cells that could be inhibited through coadministration with 100 ng/mL of PEs. Statistical analysis in Figure 4(b) suggested that almost 90% of the antisenescent activity of PEs at 100 ng/mL. In addition, IL-1*β* in the HFF-1 culture medium treated by PEs was quantified in Figure 4(c), which was significantly lower than that of H_2_O_2_-induced. These results suggested PEs made an effect on inhibition of skin senescence and inflammation, to some extent providing evidence for the link between inflammation and aging [17] and showing the prospects of PEs in skin care.

### 3.5. Anti-Inflammation of PEs in Reconstituted Skin Model

A reconstituted skin model is used to mimic dermal tissue and implies the potential role of PEs in allergic skin diseases. We used the well-established sensitive skin models: sodium lauryl sulfate (SLS)-irritated and UV-radiated models to detect the anti-inflammatory activities of PEs [71–73].  Figure S6 showed PEs decreased proinflammatory factor IL-1*α* mRNA expression more efficiently than that of positive control Dex from SLS-irritation, indicating an improvement of PEs in allergic skin. Sunlight exposure is also one of the frequent ways to encounter allergic skin and then get skin inflammation. Here, 30 J/cm^3^ of UV irradiation was used to prepare an allergic skin model. Both PEs could comparably inhibit PGE2 mRNA production compared with the nontreatment control. These results shed light on the anti-inflammation of PEs in derma mimics, which directed the possibilities of PEs' application in skin care.

### 3.6. Effects of PEs on Zebrafish

Zebrafish models have been widely used in developmental biology and drug discovery because of their low maintenance costs, transparency, and high similarity to human genomes [74, 75]. The oxidant and inflammatory models could be easily and robustly constructed and visualized in zebrafish. The copper-induced inflammatory response has been used widely because it is noninvasive compared with physical damage-induced inflammation [76, 77].

#### 3.6.1. Antioxidant Effect of PEs on Zebrafish

**Figure 5:**
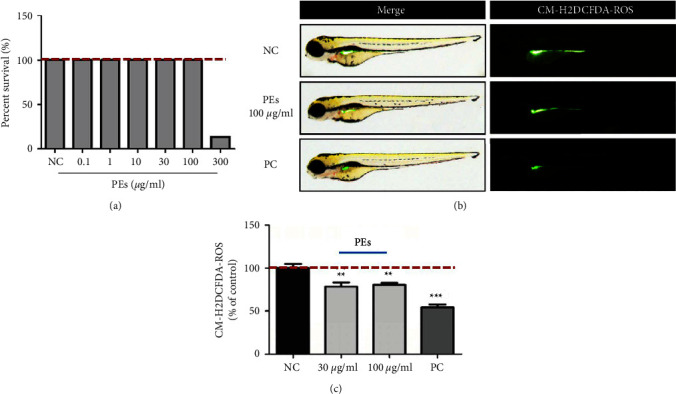
Antioxidant effect of PEs *in vivo*. Thirty zebrafish embryos were incubated either with PEs at different concentrations or Tripeptide-1 as the positive control (PC) for 24 h. After, toxicity in zebrafish embryos was determined through bright-field microscopy while the ROS levels were detected through a fluorescent dye H2DCFDA. Briefly, 20 *μ*g/mL of H2DCFDA replaced the PEs to incubate with embryos for 1 h in the dark. After washing three times, the fluorescence was observed via fluorescence microscopy (c) and the fluorescence intensity was obtained using a microplate reader (b). ^*∗*^^*∗*^*p* < 0.01, ^*∗*^^*∗∗*^*p* < 0.001 compared to the nontreatment group.

The toxicity of PEs at different concentrations in zebrafish embryos was determined by microscopy after 24 h incubation.  Figure 5(a) showed that PEs at concentrations of up to 100 *µ*g/mL has negligible toxicity on the survival rate of zebrafish embryos while 300 *µ*g/mL of PEs led to total mortality of zebrafish embryos. Hence, we treated zebrafish embryos with 30 *µ*g/mL and 100 *µ*g/mL of PEs for the following antioxidation and anti-inflammation assays. The ROS level after PEs incubation was quantified by using a fluorescence probe. The fluorescent microscopy images of zebrafish embryos in Figure 5(b) suggested fluorescence intensity significantly decreased in the presence of PEs at 100 *µ*g/mL compared with that in the nontreatment group. The bright-field microscopy indicated PEs incubation made no difference to the survival of zebrafish embryos. The fluorescence intensity is graphed in Figure 5(c), both 30 *µ*g/mL and 100 *µ*g/mL PEs evidently limited the generation of ROS *in vivo*, demonstrating the profound antioxidant effect of PEs. Furthermore, heat shock proteins (Hsp) are crucial families in protecting the cell against damage from oxidative stress. Interestingly, Figure S7 further revealed PEs at the indicated concentrations protected zebrafish embryos against oxidative damage from CuSO_4_ through the down-regulation of a stress-related gene Hsp70.

#### 3.6.2. Anti-Inflammatory Effect of PEs on Zebrafish

**Figure 6:**
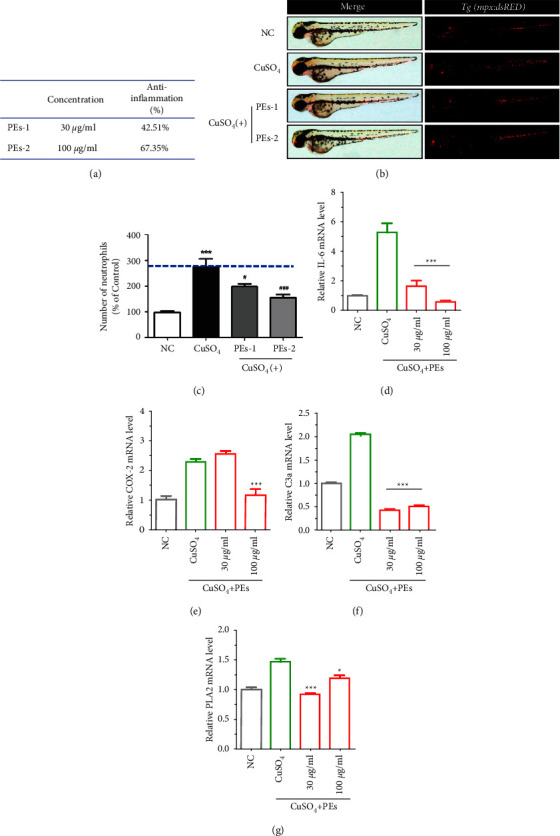
Anti-inflammatory effect of PEs against CuSO_4_-induced inflammation *in vivo*. (a) Effective concentrations of PEs and anti-inflammatory efficacies. (b) The fluorescence images of zebrafish embryos after treatment with 1 *µ*M CuSO_4_ alone or together with PEs at different concentrations. (c) The number of neutrophils in transgenic zebrafish embryos under different treatment conditions, ^*∗*^^*∗∗*^*p* < 0.001 compared with the nontreatment group, and ^#^*p* < 0.05, ^###^*p* < 0.001 compared with the group treated with CuSO_4_ alone (a-c). The expression of inflammation-related genes (IL-6, COX-2) and eicosanoid pathway-associated genes (COX-2, C3a, and PLA2) *in vivo*. The zebrafish embryos were exposed to CuSO_4_ either with PEs or not for 2h. After, embryos were collected for qRT-PCR analysis. A pool of 30 embryos per group was used. Experiments were performed in triplicates. ^*∗*^*p* < 0.05, ^*∗*^^*∗∗*^*p* < 0.001 compared to the experiment treated with CuSO_4_ alone (d-g).

The crosstalk between oxidative stress and inflammation mentioned before reminds us to study the anti-inflammatory property of PEs as well in zebrafish. Accumulation of serum copper levels that trigger oxidative stress responses and activate inflammatory responses in a dose-dependent manner. CuSO_4_-induced inflammatory responses would activate, macrophages and recruit neutrophils [78]. Therefore, the anti-inflammatory property of PEs was evaluated by monitoring the number of red-fluorescence labeled neutrophils in a neutrophil-specific transgenic zebrafish model Tg (mpx: dsRED). The red-fluorescence intensity would increase resulting from CuSO_4_.  Figure 6(a) indicated the tested concentration of PEs. The fluorescent microscopy images in Figure 6(b) revealed that coadministration with PEs at the indicated doses obviously reduced the number of red fluorescent-labeled neutrophils from the CuSO_4_ alone administration, meaning the alleviation of inflammation.  Figure 6(c) suggested PEs at 100 *μ*g/mL decreased by around 67% of the number of neutrophils compared with the treatment of CuSO_4_ alone. We subsequently quantified the levels of inflammation-related genes (Primer was listed in Table S2). However, Figures 6(d)–6(g) suggested after CuSO_4_ exposure, PEs at 100 *μ*g/mL excellently downregulated the levels of IL-6, complement C3a, and phospholipase A2 (PLA2) mRNA, which are involved in MAPK-mediated inflammatory signaling pathway.

## 4. Discussion

The human derma is an important organ for protecting the human body from the invasion of external stimuli. Nowadays, people are more concerned about the appearance of the skin. Most skin disorders are chronic and inflammatory, which result in superficial skin changes and produce crosstalk with other pathophysiological processes. Moreover, inflammatory cytokines are members of senescence-associated secretory phenotypes (SASPs) secreted through senescent cells, meaning inflammatory response might accelerate the development of senescence. Thus, inhibition of skin inflammation seems to be an alternative way to cure skin disorders, aging, and inflammation-related illness. Traditional natural plants have been considered desirable candidates for medical and cosmeceutical ingredients because of their safety and efficacy *in vitro* and *in vivo*. *Physalis alkekengi* L. belongs to the Solanaceae family distributed widely in China and is popular both in food and folk medicine. However, the anti-inflammatory activity of *Physalis alkekengi* L. extracts (PEs) has been most exhibited in immune cells, the studies of the anti-inflammatory and antisenescent activities of PEs in human skin cells remain scarce.

Our study showed the protective effects of PEs from LPS-induced damage through inhibition of inflammation-related enzymes and gene expression not only in murine macrophage RAW264.7 but also in one skin cell line, keratinocyte HaCaT. Interestingly, PEs also attenuated the expression of senescence-associated marker SA-*β*-gal and proinflammatory factor IL-1*β* in skin fibroblast cells HFF-1 induced by D-gal and H_2_O_2_, which to some extent emphasized the interdependent relationship between inflammation and aging. Importantly, the study also initiatively illustrated the anti-inflammatory properties of PEs in SLS-stimulated and UV-radiated 3D reconstructed human skin models for allergy research, implying the impact of PEs on allergic skin diseases. Here, *in vivo* anti-inflammation of PEs has also been demonstrated in the copper-sulfate-induced zebrafish embryos model. These robust results provided a strong basis for the promising application of PEs in the fields of anti-inflammatory medicine and cosmetics.

**Figure 7:**
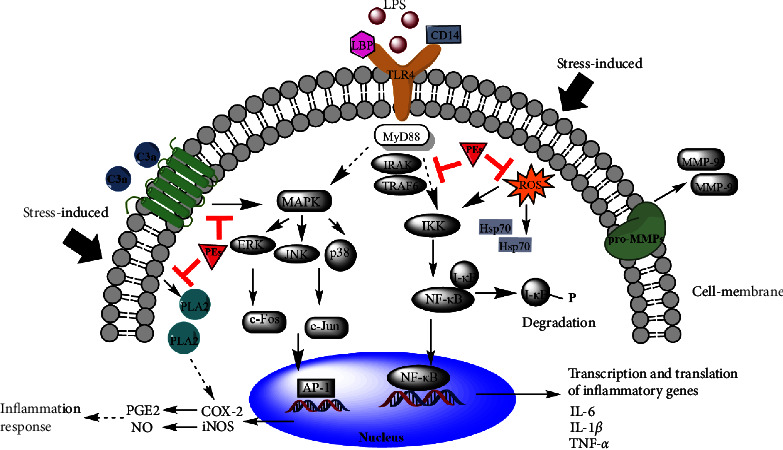
Proposed roles of PEs in the suppression of LPS-induced and oxidative stress-induced inflammatory pathways.

With respect to the mechanisms of PEs in response to inflammation and oxidative stress, we envisaged the possible roles of PEs in the anti-inflammatory and antioxidant pathways in Figure 7. Taken together, LPS molecules together with LPS binding protein (LBP) bound to CD14 were transferred to toll-like receptor 4 (TLR4)-MyD88 complex to initiate the inflammatory pathways, although the exact roles of LBP and CD14 in the recognition of LPS remain elusive. MAPK and IKK, the downstream proteins of MyD88 were activated in the subsequent MAPK/AP-1 and IKK/NF-*κ*B signaling with the production of proinflammatory cytokines such as IL-6, IL-1*β*, and TNF-*α*. As for the stimuli of oxidative stress, on the one hand, oxidative stress from the treatment of H_2_O_2_ or copper sulfate increased the generation of intracellular ROS to activate the NF-*κ*B signaling, on the other hand, stress-activated the metabolizing enzymes such as phospholipase A2 (PLA2) and COX-2 for the degradation of membrane lipids to a final product PGE2. Therefore, oxidative damage exacerbated the inflammation response. On the basis of the study, PEs directly reduced the ROS levels, which partly inhibited the NF-*κ*B signaling. Moreover, PEs might suppress MAPK-mediated inflammation through the down-regulation of PLA2 and complement C3a levels. However, further studies are required to interpret whether the expression of mRNA is consistent with the production of corresponding proteins due to transcriptional and post-translational modifications. Furthermore, pieces of evidence are needed to identify the exact targets of PEs in the anti-inflammatory pathways and the roles of PEs (besides inhibition of MMP-9) in cell senescence which could broaden the application of PEs in skin care products.

## 5. Conclusions

In summary, this work showed the anti-inflammatory and antioxidant effects of traditional herbal medicines PEs *in vitro* and *in vivo*. More importantly, this paper explored the antisenescent activity of PEs through suppression of SASPs which revealed, to some extent, the relationship between inflammation and senescence. In the end, we illustrated the possible roles of PEs in two main inflammation-related signaling pathways, as summarized in Figure 7. Accordingly, the robust data shed light on PEs as a promising anti-inflammatory and antisenescent ingredient in skincare-associated products. However, further studies are needed to isolate the biologically functional compounds involved in PEs and identify the exact targets to evaluate their activities.

## Data Availability

All data generated or analyzed during this study are included in this published article. Raw data are available from the corresponding author upon reasonable request.

## Supplementary Materials

Supplementary Materials Table S1. primers used to analyze the expression of inflammatory genes in RAW264.7. Table S2. Primers used to analyze the expression of inflammatory genes in zebrafish. Figure S1: bright-field microscopy images of RAW264.7 treated by PEs at 50, 100 *μ*g/mL for 1 h prior to incubation with LPS (1 *μ*g/mL) for 24 h. Treatment of LPS plus Dex at 10 *μ*g/mL was a positive control while incubation of PEs at 100 *μ*g/mL alone was a negative control. The scale bar represents 20 *µ*m. Figure S2: cytotoxicity of PEs at various concentrations for 12, 24, and 36 h incubation in keratinocytes HaCaT. Cell viability was measured by MTT assay. Three independent experiments (triplicate for each) were performed. ^*∗*^^*∗*^*p* < 0.01 compared to the nontreatment group. Figure S3: protective effect of PEs from LPS-damage on HaCaT. Cells were incubated either with PEs alone at different does or not for 24 h, then observed using bright-field microscopy (a). Cells were incubated with PEs for 12 h prior to treatment with 10 *μ*g/mL of LPS for another 12 h. Then, cell viability was measured via MTT assay (b). Triplicate was done for each experiment group. ^*∗*^*p* < 0.05, ^*∗*^^*∗*^*p* < 0.01 compared to the LPS-induced group while ^##^*p* < 0.01 compared to control. The scale bar represents 20 *µ*m. Figure S4: anti-inflammatory effects of PEs on TNF-*α* (a), IL-6(b), and IL-1*β* (c) expression in LPS-induced HaCaT culture medium using enzyme-linked immunosorbent assay (ELISA). Cells were treated with PEs at the concentrations of 25, 50, and 100 *μ*g/mL for 12 h before incubation with LPS (10 *μ*g/mL) for another 12 h. Triplicate was conducted in the experiments. ^*∗∗*^*p* < 0.01 compared to the cells treated with LPS alone, ^##^*p* < 0.01 compared with control. Figure S5: antioxidant effect of PEs on HaCaT. Cells were pretreated with PEs at different concentrations for 2 h, then incubated with 800 *μ*M of H_2_O_2_ for another 2 h. The level of reactive oxygen species (ROS) was quantified immediately using a membrane-permeable probe DCFH-DA and flow Cytometry (FCM) (a). The cytotoxicity of PEs at 100 ng/mL in HaCaT was measured through LDH release as well (b) Triplicate was conducted in the experiments. ^*∗*^*p* < 0.05,  ^*∗∗*^*p* < 0.01,  ^*∗∗∗*^*p* < 0.001 compared to the cells treated with H_2_O_2_ alone. Figure S6: anti-inflammation effect of PEs on IL-1*α* (a) and PGE2 (b) in SLS-irritated or UV-radiated 3D skin models using enzyme-linked immunosorbent assay (ELISA). The skin was transferred into a 6-well plate and treated either with 0.3% SLS or 30J/cm^3^ UV for 2 h, followed by 1 mg/mL of PEs or Dex incubation for another 20 h.  ^*∗∗∗*^*p* < 0.001,  ^*∗∗∗∗*^*p* < 0.0001, compared to the non-SLS- and non-UV-stimulated skin but still treated with PEs or Dex. Figure S7: the expression of the antioxidant gene (Hsp70) *in vivo*. Thirty zebrafish embryos were exposed to CuSO_4_ either with PEs or not for 2 h. After, embryos were collected for qRT-PCR analysis. Experiments were performed in triplicates.  ^*∗∗∗*^*p* < 0.001 compared to the experiment treated with CuSO_4_ alone.
