# Supplementary material for: Anti-Inflammatory and Antioxidant Properties of Physalis alkekengi L. Extracts In Vitro and In Vivo: Potential Application for Skin Care
Source: Evid Based Complement Alternat Med. 2022 Oct 19;2022:7579572. doi: 10.1155/2022/7579572 (PMC9605834; doi:10.1155/2022/7579572)
Supplement: Supplementary Materials — Table S1. primers used to analyze the expression of inflammatory genes in RAW264.7. Table S2. Primers used to analyze the expression of inflammatory genes in zebrafish. Figure S1: bright-field microscopy images of RAW264.7 treated by PEs at 50, 100 μg/mL for 1 h prior to incubation with LPS (1 μg/mL) for 24 h. Treatment of LPS plus Dex at 10 μg/mL was a positive control while incubation of PEs at 100 μg/mL alone was a negative control. The scale bar represents 20 µm. Figure S2: cytotoxicity of PEs at various concentrations for 12, 24, and 36 h incubation in keratinocytes HaCaT. Cell viability was measured by MTT assay. Three independent experiments (triplicate for each) were performed. ∗∗p < 0.01 compared to the nontreatment group. Figure S3: protective effect of PEs from LPS-damage on HaCaT. Cells were incubated either with PEs alone at different does or not for 24 h, then observed using bright-field microscopy (a). Cells were incubated with PEs for 12 h prior to treatment with 10 μg/mL of LPS for another 12 h. Then, cell viability was measured via MTT assay (b). Triplicate was done for each experiment group. ∗p < 0.05, ∗∗p < 0.01 compared to the LPS-induced group while ##p < 0.01 compared to control. The scale bar represents 20 µm. Figure S4: anti-inflammatory effects of PEs on TNF-α (a), IL-6(b), and IL-1β (c) expression in LPS-induced HaCaT culture medium using enzyme-linked immunosorbent assay (ELISA). Cells were treated with PEs at the concentrations of 25, 50, and 100 μg/mL for 12 h before incubation with LPS (10 μg/mL) for another 12 h. Triplicate was conducted in the experiments. ∗∗p < 0.01 compared to the cells treated with LPS alone, ##p < 0.01 compared with control. Figure S5: antioxidant effect of PEs on HaCaT. Cells were pretreated with PEs at different concentrations for 2 h, then incubated with 800 μM of H2O2 for another 2 h. The level of reactive oxygen species (ROS) was quantified immediately using a membrane-permeable probe DCFH-DA and flow Cytome [file 7579572.f1.docx]

**Supporting information**

**Table S1**. Primers used to analyze the expression of inflammatory genes in RAW264.7

| Gene Name | Forward and Reverse Primer sequences (5’-3’) | Resources |
| --- | --- | --- |
| β-actin | *Fwd*: TGT TTG AGA CCT TCA ACA CC  *Rev*: AGT CTG TCA GGT CCC GGC C | Murine |
| IL-6 | *Fwd*: CTG CAA GAG ACT TCC ATC CAG  *Rev*: AGT GGT ATA GAC AGG TCT GTT GG | Murine |
| IL-10 | *Fwd*: CTT ACT GAC TGG CAT GAG GAT CA  *Rev*: GCA GCT CTA GGA GCA TGT GG | Murine |
| IL-α | *Fwd*: TCT ATG ATG CAA GCT ATG GCT CA  *Rev*: CGG CTC TCC TTG AAG GTG A | Murine |
| IL-1β | *Fwd*: GAA ATG CCA CCT TTT GAC AGT G  *Rev*: TGG ATG CTC TCA TCA GGA CAG | Murine |
| TNF-α | *Fwd*: CTG AAC TTC GGG GTG ATC GG  *Rev*: GGC TTG TCA CTC GAA TTT TGA GA | Murine |
| NOS | *Fwd*: GTT CTC AGC CCA ACA ATA CAA GA  *Rev*: GTG GAC GGG TCG ATG TCA C | Murine |
| COX-2 | *Fwd*: GAA GTC TTT GGT CTG GTG CGT G  *Rev*: GTC TGC TGG TTT GGA ATA GTT GC | Murine |
| PGE2 | *Fwd*: CAG CTC GGT GAT GTT CTC GG  *Rev*: GAG CAC CAA TTC CGT TAC CAG | Murine |

**Table S2**. Primers used to analyze the expression of inflammatory genes in zebrafish

| **Gene Name** | **Forward and Reverse Primer sequences (5’-3’)** | **Resources** |
| --- | --- | --- |
| β-actin | *Fwd*: CCC CAT TGA GCA CGG TAT TG  *Rev*: ATA CAT GGC AGG GGT GTT GA | Zebrafish |
| IL-6 | *Fwd*: ACG GAA AGA TGT CTA ACG C  *Rev*: ATA GGG AAG TGC TGG ATG | Zebrafish |
| Hsp70 | *Fwd*: CAA CGT GCT GAT CTT TGA CC  *Rev*: TCC TCT TGG CTC GTT CAC AT | Zebrafish |
| COX-2 | *Fwd*: ACA GAT GCG CTA CCA GTC TT  *Rev*: CCC ATG AGG CCT TTG AGA GA | Zebrafish |
| C3a | *Fwd*: GTA CGA GGC GAA CAA CTG GA  *Rev*: CAT CAT ACG CCG CAG CTT TC | Zebrafish |
| PLA2 | *Fwd*: TCA TGT CTC CTG GGC TGT TT  *Rev*: CCA GCT CCT CCT CCA TAG TG | Zebrafish |


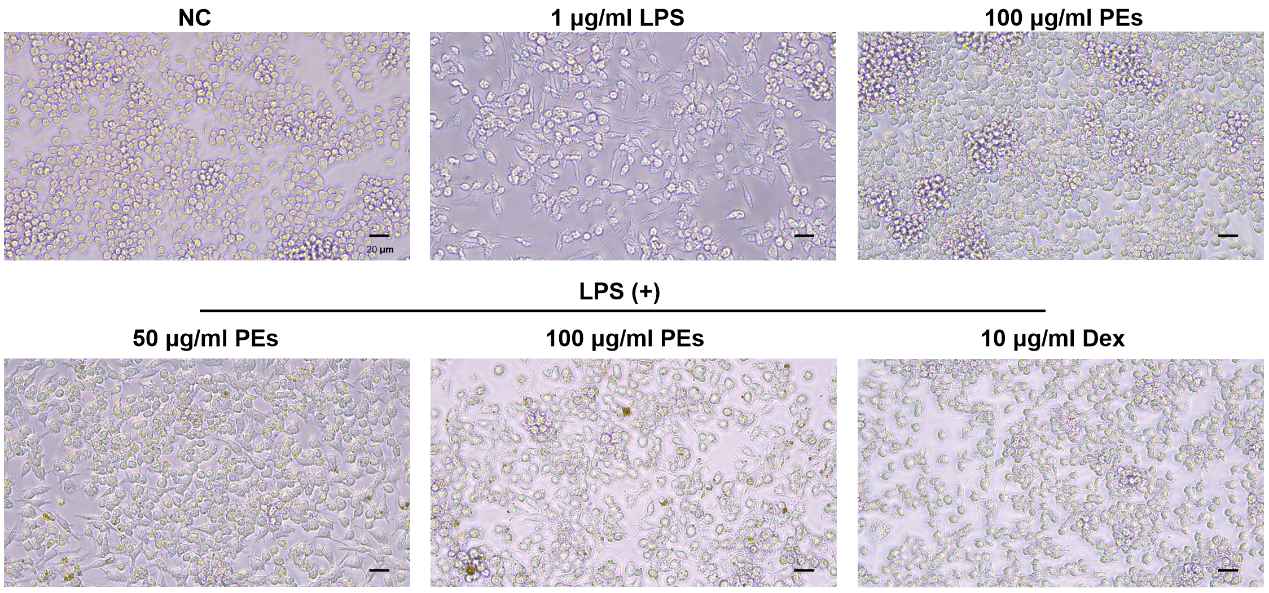


Figure S1: Bright-field microscopy images of RAW264.7 treated by PEs at 50, 100 μg/mL for 1 h prior to incubation with LPS (1 μg/mL) for 24 h. Treatment of LPS plus Dex at 10 μg/mL was as positive control while incubation of PEs at 100 μg/mL alone was as negative control. Scale bar represents 20 µm.


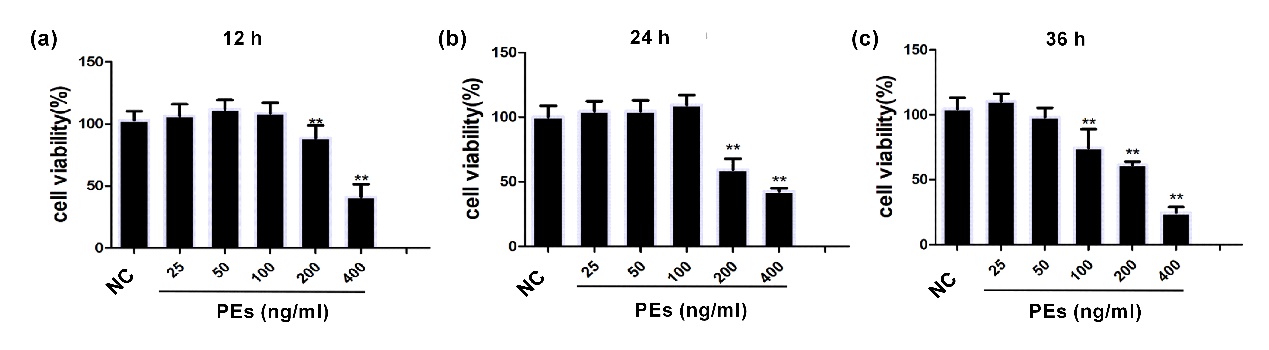


Figure S2: Cytotoxicity of PEs at various concentrations for 12, 24 and 36 h incubation in keratinocytes HaCaT. Cell viability was measured by MTT assay. Three independent experiments (triplicate for each) were performed. **p < 0.01 compared to the non-treatment group.


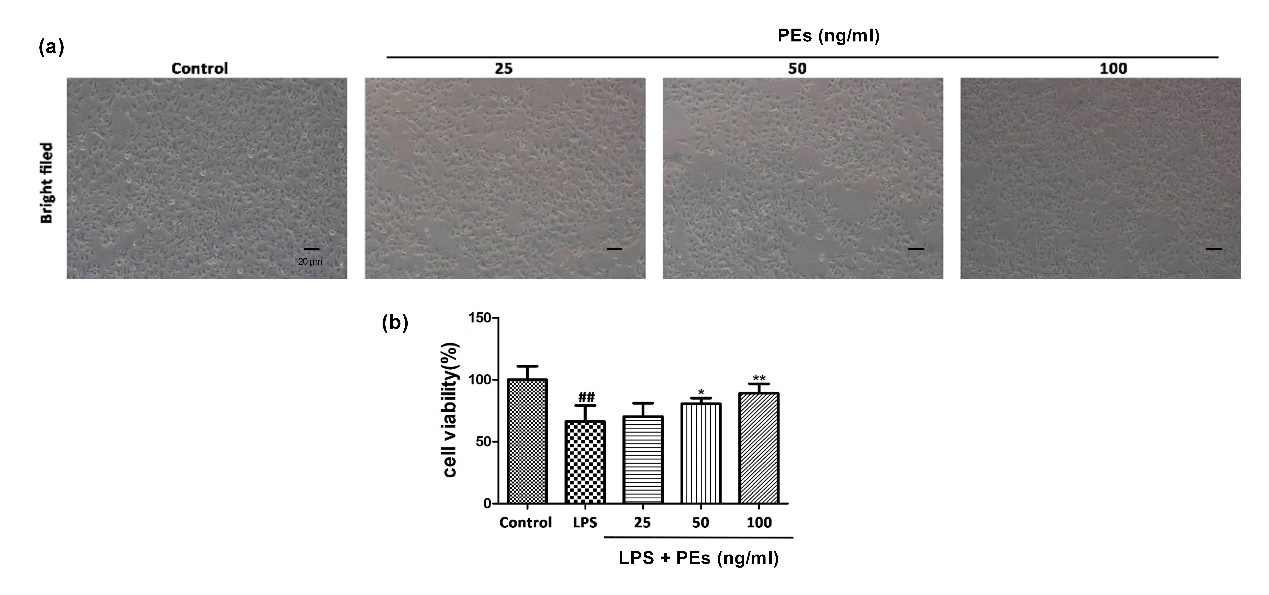


Figure S3: Protective effect of PEs from LPS-damage on HaCaT. Cells were incubated either with PEs alone at different does or not for 24 h, then observed using bright-field microscopy (a). Cells were incubated with PEs for 12 h prior to treatment with 10 μg/mL of LPS for more 12 h. Then, cell viability was measured via MTT assay (b). Triplicate was done for each experiment group. *p < 0.05, **p < 0.01 compared to the LPS-induced group while ^##^p < 0.01 compared to control. Scale bar represents 20 µm.


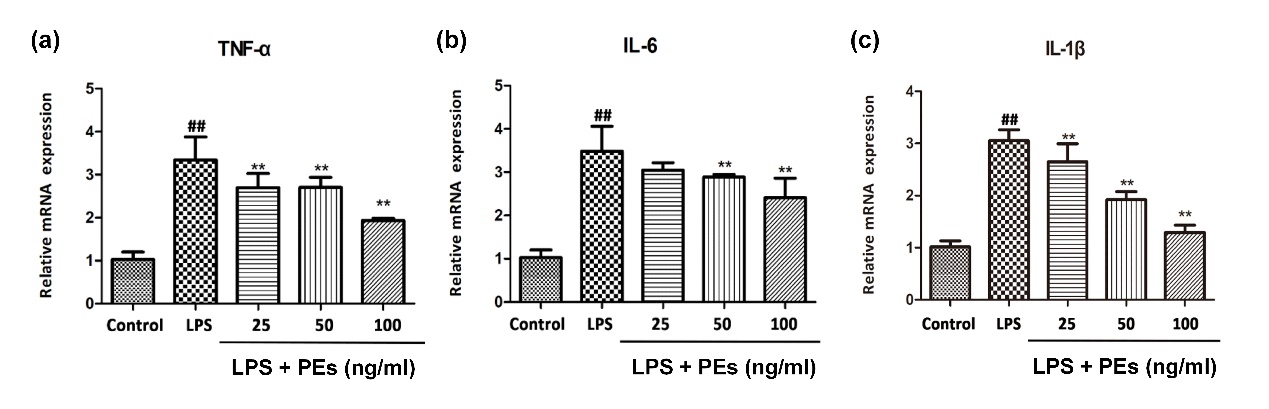


Figure S4: Anti-inflammatory effects of PEs on TNF-α (a), IL-6(b) and IL-1β (c) expression in LPS-induced HaCaT culture medium using enzyme-linked immunosorbent assay (ELISA). Cells were treated with PEs at the concentrations 25, 50, 100 μg/mL for 12 h before incubation with LPS (10 μg/mL) for another 12 h. Triplicate was conducted in the experiments. **p < 0.01 compared to the cells treated with LPS alone, ^##^p < 0.01 compared with control.


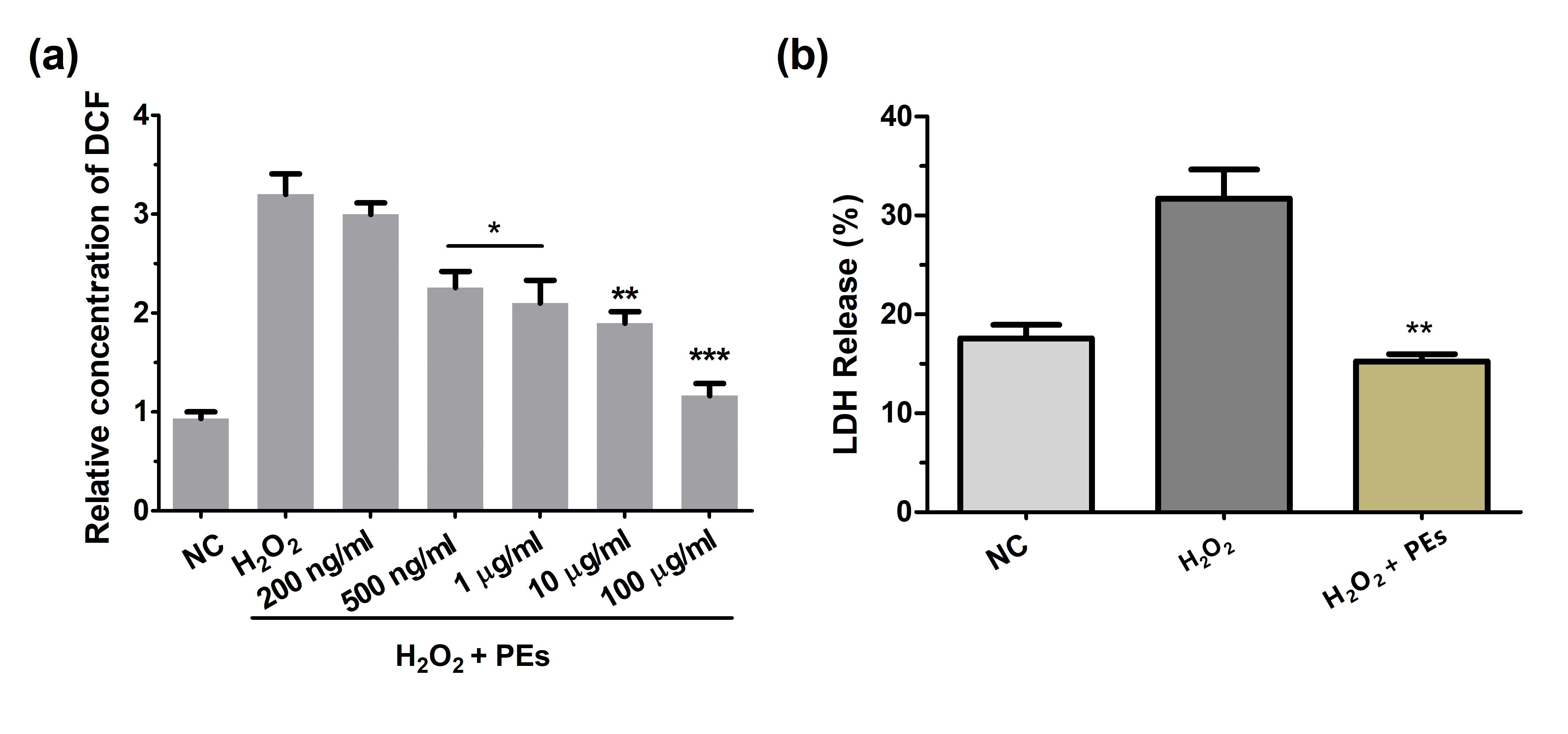


Figure S5: Anti-oxidant effect of PEs on HaCaT. Cells were pretreated with PEs at different concentrations for 2 h, then incubated with 800 μM of H_2_O_2_ for another 2 h. The level of reactive oxygen species (ROS) was quantified immediately using a membrane-permeable probe DCFH-DA and flow Cytometry (FCM) (a). The cytotoxicity of PEs at 100 ng/mL in HaCaT was measured through LDH release as well (b) Triplicate was conducted in the experiments. *p < 0.05, **p < 0.01, ***p < 0.001 compared to the cells treated with H_2_O_2_ alone.


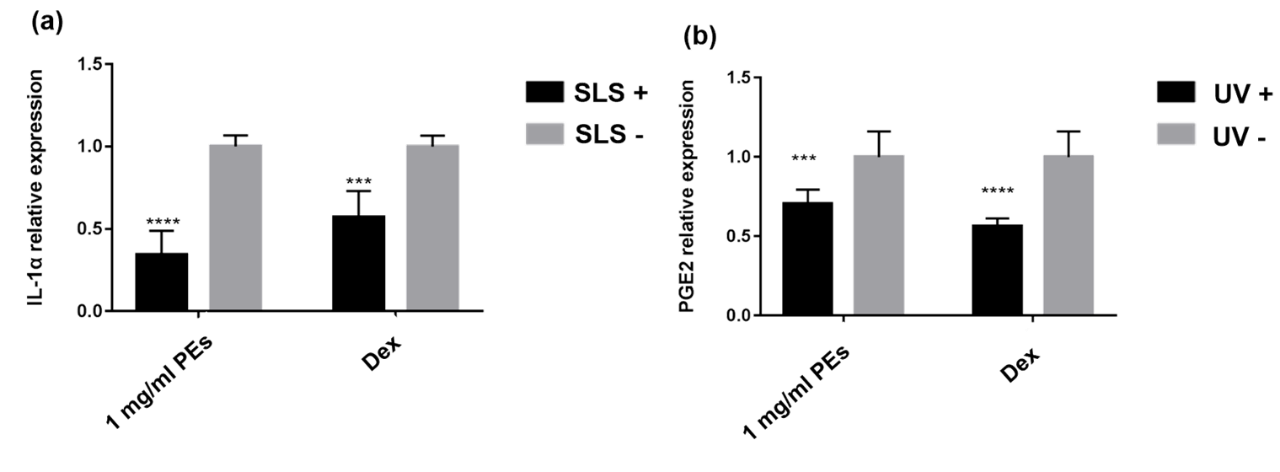


Figure S6: Anti-inflammation effect of PEs on IL-1α (a) and PGE2 (b) in SLS-irritated or UV-radiated 3D skin models using enzyme-linked immunosorbent assay (ELISA). Skin were transferred into a 6-well plate and treated either with 0.3% SLS or 30J /cm^3^ UV for 2 h, followed by 1 mg/mL of PEs or Dex incubation for another 20 h. ***p < 0.001, ****p < 0.0001, compared to the non-SLS- and non-UV-stimulated skin but still treated with PEs or Dex.





Figure S7: The expression of the antioxidant gene (Hsp70) *in vivo*. Thirty zebrafish embryos were exposed to CuSO_4_ either with PEs or not for 2h. After, embryos were collected for qRT-PCR analysis. Experiments were performed in triplicates. ***p < 0.001 compared to the experiment treated with CuSO_4_ alone.
